# Sex disparities in diagnostic workup of benign pancreaticobiliary disease before endoscopic retrograde cholangiopancreatography

**DOI:** 10.1016/j.igie.2025.03.007

**Published:** 2025-03-31

**Authors:** Sarah Dwyer Holland, Garrett Weskamp, Gabriel Brutico, Ford Holland, David L. Diehl, Anjuli Luthra, Shaffer Mok

**Affiliations:** 1 Department of Gastroenterology & Hepatology, Brown University, Providence, Rhode Island, USA; 2 Department of Medicine, University of Colorado, Aurora, Colorado, USA; 3 Department of Medicine, St. Luke's University Hospital, Bethlehem, Pennsylvania, USA; 4 Independent Researcher, Providence, Rhode Island, USA; 5 Department of Gastroenterology, Geisinger Medical Center, Danville, Pennsylvania, USA; 6 Department of Gastrointestinal Oncology, H. Lee Moffitt Cancer Center, Tampa, Florida, USA

## Abstract

**Background and Aims:**

Women face diagnostic delays in the workup of many diseases. We investigated whether there are sex-based disparities in the diagnostic workup of patients with benign pancreaticobiliary pathologies requiring endoscopic retrograde cholangiopancreatography (ERCP).

**Methods:**

We conducted a multicenter retrospective cohort study including patients undergoing ERCP from December 2017 to 2021 for benign pancreaticobiliary pathologies. We assessed differences between males and females in days from presentation to ERCP, number of healthcare visits before ERCP, symptoms, and diagnostic workup at first visit.

**Results:**

One hundred twenty-eight patients (68 female patients) were identified. Eighteen percent of male patients initially presented with fever or chills versus 6% of female patients (*P* = .03), but otherwise there were no differences in symptoms. There was no difference between sexes in number of encounters or days from presentation until ERCP (*P* = .5). Significant differences occurred in the initial diagnostic evaluation. Male patients had liver chemistry testing in 98% of initial visits versus 85% of female patients (*P* = .01), and 95% had imaging ordered at first visit versus 82% of female patients (*P* = .03). Excluding febrile patients, male patients remained more likely to have laboratory (*P* = .02) and imaging evaluations (*P* = .05) at the initial visit. Thirteen female patients (19%) had an inadequate workup at the first visit for benign pancreaticobiliary conditions compared with 3 male patients (5%, *P* = .02).

**Conclusions:**

We did not observe sex-based delays in time from presentation to ERCP in our sample; however, women were less likely to have adequate workup for benign pancreaticobiliary conditions at the first visit.

Differences between men and women in the epidemiology, pathogenesis, and presentation of many diseases have been well established.^1^ However, an increasing number of studies had shown that sex and gender biases may be implicated in delayed diagnosis of diseases in women. For example, women have a significantly longer time between first medical contact and myocardial infarction diagnosis^2^ and are more likely to have reperfusion delays than men,^3^ which does not appear to be explained by an increased rate of atypical presentation.^4^ Moreover, women are less likely to be diagnosed with asthma despite a generally more severe phenotype,^5^ have delayed diagnosis of appendicitis compared with men,^6^ and have a longer period from symptom onset to inflammatory bowel disease diagnosis with a higher likelihood of misdiagnosis.^7^

When considering potential disparities in the workup of patients requiring endoscopic retrograde cholangiopancreatography (ERCP), it is important to acknowledge differences in pathophysiology and clinical presentation between men and women. Gallstones, which can cause choledocholithiasis and cholangitis, have a 2- to 3-fold higher incidence in women compared with men.^8^ Moreover, 1 study found that men requiring cholecystectomy had more complicated cases of cholecystitis compared with women, with longer intraoperative times and higher conversion rates from laparoscopic to open laparotomy.^9^

Whether there are differences between sexes in the initial workup of benign pancreaticobiliary symptomatology is unknown. Decreasing time to ERCP can reduce morbidity and mortality from ascending cholangitis.^10^ In this study, we aimed to identify differences between men and women in the diagnostic workup of benign pancreaticobiliary diseases that eventually require ERCP.

## Methods

In this multicenter, retrospective cohort study, we included patients who underwent ERCP from 2017 to 2021 at 2 tertiary care centers (Geisinger Health System in Danville, Pennsylvania, USA, and University Hospitals in Cleveland, Ohio, USA) and 3 community institutions in the Geisinger Health System and University Hospitals System. Patients undergoing ERCP were identified using an endoscopic database at both institutions. Included patients were older than 17 years with greater than 6 months of notes in the health record preceding ERCP and with benign pancreaticobiliary pathologies including common bile duct stone, pancreatic duct stone, benign papillary stenosis, ampullary adenoma, benign biliary stricture, and gallstone pancreatitis. Exclusion criteria were prior ERCP, previous history of any pancreaticobiliary condition, history of liver transplant, or malignancy-related biliary pathology.

After patients were identified, demographic data and patient-reported symptoms on the day of ERCP were documented. Using each institution’s electronic medical record, we conducted a manual review of every visit to the primary care provider office, urgent care, or emergency department in the previous 6 months. If a given visit was for the symptom noted on the day of ERCP, we collected data including whether aspartate aminotransferase and alanine aminotransferase, bilirubin, alkaline phosphatase, lipase, amylase, or any imaging including ultrasound, computed tomography, magnetic resonance imaging, or hepatobiliary iminodiacetic acid scan were performed. We also noted whether these study results were normal or abnormal. If a patient had more than 1 visit before ERCP, we documented what the presumed diagnosis was from the first visit. Referral to the emergency department from a primary care or urgent care visit was counted as 1 visit, and laboratory and imaging testing collected at both locations was recorded. We defined complete workup for pancreaticobiliary pathologies as having both standard liver chemistries and abdominal imaging at the first visit.

Primary outcomes assessed were the number of days from the first visit until ERCP and number of healthcare visits until ERCP. Secondary outcomes were frequency of appropriate laboratory and imaging workup at the first visit.

We report cohort age, sex, race, and ethnicity using descriptive statistics. We assessed differences between sexes in chief complaint, diagnosis, number of visits and number of days until ERCP, laboratory tests, and imaging at the initial visit. The number of visits until ERCP from initial presentation was stratified as 1 or >1 visit. Because chief complaints can affect perceived urgency in case workups, we additionally assessed the frequency of chief complaints between males and females. Finally, we analyzed differences between the sexes in the frequency of laboratory test or imaging test ordered at the first visit. For each variable, a Pearson χ^2^ test, Fisher exact test, or Wilcoxon rank sum test was performed to determine the difference between the male and female cohorts and calculate statistical significance. All tests were performed with an alpha level set at 0.05. We report median (IQR) or mean (standard deviation [SD]) for all descriptive statistics.

## Results

One hundred twenty-eight patients (68 female patients and 60 male patients) met the study criteria. Most patients were white (89%) and non-Hispanic (98%) (Table 1). The average age of patients was 72 years (SD, 19), and there was no significant difference in age between male and female patients (*P* = .09). Only 2 patients in this cohort had outpatient ERCPs: the first occurred after inpatient ERCP was unsuccessful and the second occurred after an outpatient workup. For both sexes, there was no difference in the prevalence of abdominal pain, nausea or vomiting, bloating, weight loss, jaundice, fatigue, or altered mental status (Table 2). However, 18% of male patients compared with 5.9% of female patients reported fevers or chills (*P* = .03). Of patients who did have laboratory testing at their first visit, 71% of female patients had elevated bilirubin levels compared with 80% male patients (*P* = .3), and 69% of female patients had elevated alkaline phosphatase values compared with 75% of men (*P* = .5).

**Table 1:** Patient characteristics

	Men (n = 60)	Women (n = 68)	*P* value∗
Age, y	72 (63-80)	71 (42-78)	.090
Race			.5
White	52 (87%)	62 (91%)	
African American	4 (6.7%)	5 (7.4%)	
Other	1 (1.7%)	0 (0%)	
Unknown	3 (5.0%)	1 (1.5%)	
Ethnicity			.7
Non-Hispanic	59 (98%)	67 (99%)	
Hispanic	0 (0%)	1 (1.5%)	
Unknown	1 (1.7%)	0 (0%)	
Location of first visit			.2
Emergency department	50 (83%)	56 (82%)	
Primary care provider	10 (17%)	8 (12%)	
Urgent care	0 (0%)	4 (5.9%)	

Values are median (interquartile range [IQR]) or n (%).

∗ Wilcoxon rank sum test; Fisher exact test.

**Table 2:** Patient-reported symptoms at time of ERCP and diagnosis from ERCP report

	Men (n = 60)	Women (n = 68)	*P* value∗
Symptom			
Abdominal pain	50 (83%)	61 (90%)	.3
Nausea or vomiting	34 (57%)	49 (72%)	.069
Jaundice	14 (23%)	9 (13%)	.14
Fatigue	8 (13%)	7 (10%)	.6
Subjective fever or chills	11 (18%)	4 (5.9%)	.029
Weakness	6 (10%)	8 (12%)	.7
Altered mental status	2 (3.3%)	8 (12%)	.10
Weight loss	6 (10%)	2 (2.9%)	.15
Bloating	0 (0%)	2 (2.9%)	.5
Diagnosis			
Common bile duct stone	49 (82%)	57 (84%)	.7
Gallstone pancreatitis	10 (17%)	8 (12%)	.4
Benign biliary stricture	7 (12%)	6 (8.8%)	.6
Benign papillary stenosis	4 (6.7%)	8 (12%)	.3
Pancreatic duct stone	2 (3.3%)	0 (0%)	.2
Papillary adenoma	0 (0%)	1 (1.5%)	>.9

Values are n (%).

*ERCP*, Endoscopic retrograde cholangiopancreatography.

∗ Pearson χ^2^ test; Fisher exact test.

There was no statistical difference between male and female patients in diagnosis made during ERCP (Table 2). The most common diagnosis during ERCP was choledocholithiasis, occurring in 84% of female patients and 82% of male patients (*P* = .7). Seventeen female patients (25%) underwent ERCP after more than 1 visit from their initial presentation compared with 11 male patients (18%, *P* = .4), and the median total number of days until ERCP was the same for male and female patients (*P* = .5) (Table 3).

**Table 3:** Median number of days from first visit until ERCP and percentage of patients with more than 2 visits before ERCP

	Men (n = 60)	Women (n = 68)	*P* value∗
Time to intervention			
Time from presentation to ERCP, days	2 (1-5%)	2 (1-4%)	.5
More than 1 visit	11 (18%)	17 (25%)	.4
Workup at first visit			
Standard liver chemistries	59 (98%)	58 (85%)	.009
Lipase	46 (77%)	44 (65%)	.14
Amylase	9 (15%)	5 (7.4%)	.2
Abdominal imaging	57 (95%)	56 (82%)	.026

Values are median (IQR) or n (%).

*ERCP*, Endoscopic retrograde cholangiopancreatography.

∗ Wilcoxon rank sum test; Pearson χ^2^ test.

Female patients were less likely to have standard liver chemistries tested at the first visit (*P* = .01). Abdominal imaging at the first visit occurred in 95% of male patients compared with 82% of female patients, which was a statistically significant difference (*P* = .03) (Table 3). Notably, even after stratification by fever, female patients without fever were still less likely to have standard liver chemistries tested (84% vs 98%, respectively; *P* = .02) or any abdominal imaging (81% vs 94%, respectively; *P* = .05) than male patients without fever.

Table 4 describes laboratory and imaging workup stratified by location of first visit. Of patients who presented to the emergency department for their first visit, 93% of female patients and 100% of male patients had standard liver chemistries tested, which was not a significant difference (*P* = .12). Although there were not enough cases to make a reliable estimate, the data suggest that female patients outside the emergency department did not receive standard liver chemistry (50% vs 90%, respectively; *P* = .07) and imaging workup (42% vs 80%, respectively; *P* = .1) at the same rate as male patients.

**Table 4:** Percentage of patients with laboratory and imaging workup at first visit by presenting location

	Emergency department (n = 106)			Non–emergency department (n = 22)		
	Men (n = 50)	Women (n = 56)	*P* value∗	Men (n = 10)	Women (n = 12)	*P* value†
Standard liver chemistries	50 (100%)	52 (93%)	.12	9 (90%)	6 (50%)	.074
Lipase	39 (78%)	39 (70%)	.3	7 (70%)	5 (42%)	.2
Amylase	8 (16%)	5 (8.9%)	.3	1 (10%)	0 (0%)	.5
Abdominal imagining	49 (98%)	51 (91%)	.2	8 (80%)	5 (42%)	.10

Values are n (%).

∗ Fisher exact test; Pearson χ^2^ test.

† Fisher exact test.

Thirteen female patients (19%) had an incomplete workup for pancreaticobiliary pathologies compared with 3 male patients (5%) (*P* = .02). Table 5 outlines the characteristics, presenting location, workup, and diagnosis at the first visit for each of these patients. Two female patients and no male patients were diagnosed with anxiety at their first visit.

**Table 5:** Characteristics, presenting location, workup, and diagnosis at first visit for patients with incomplete workup for pancreaticobiliary symptoms

Age (y)	Sex	Presenting location	Symptoms	Liver serologies at first visit	Imaging at first visit	Diagnosis at first visit	No. of visits before diagnosis	No. of days before ERCP	Diagnosis at ERCP
70	F	ED	Abdominal pain and nausea/vomiting	No	No	Unspecified abdominal pain	2	4	Choledocholithiasis
41	F	ED	Abdominal pain and nausea/vomiting	No	No	Anxiety	3	4	Benign papillary stenosis
33	F	UC	Abdominal pain and nausea/vomiting	No	No	Unspecified abdominal pain	2	2	Choledocholithiasis and gallstone pancreatitis
35	F	UC	Abdominal pain and nausea/vomiting	No	No	Unspecified abdominal pain	2	2	Choledocholithiasis
19	F	ED	Abdominal pain	No	No	Anxiety	3	80	Choledocholithiasis
75	F	ED	Abdominal pain, weakness, fatigue, encephalopathy	No	No	Hypoglycemia	2	2	Choledocholithiasis, benign papillary stricture, gallstone pancreatitis
61	F	PCP	Abdominal pain	No	No	Unspecified abdominal pain	2	31	Choledocholithiasis
60	F	PCP	Nausea	No	No	Dark urine	2	14	Choledocholithiasis, benign papillary stenosis
42	F	UC	Abdominal pain, nausea/vomiting, weakness, fatigue	No	No	Unspecified abdominal pain	5	20	Choledocholithiasis
35	F	PCP	Abdominal pain, nausea/vomiting	No	Yes	Unspecified abdominal pain	2	2	Choledocholithiasis
82	F	ED	Abdominal pain, nausea/vomiting	Yes	No	Elevated transaminases, chronic obstructive pulmonary disease exacerbation	4	50	Choledocholithiasis, papillary adenoma
43	F	UC	Abdominal pain, nausea/vomiting, weight loss	Yes	No	Unspecified abdominal pain	2	11	Choledocholithiasis
72	F	PCP	Abdominal pain	Yes	No	Unspecified abdominal pain	2	23	Choledocholithiasis
75	M	PCP	Abdominal Pain	No	No	Unspecified abdominal pain	2	30	Choledocholithiasis
55	M	PCP	Abdominal Pain	Yes	No	Angina	3	41	Choledocholithiasis, gallstone pancreatitis
73	M	ED	Weight loss	Yes	No	Weight loss, elevated transaminases, elevated bilirubin	3	22	Benign biliary stricture

*ED*, Emergency department; *ERCP*, endoscopic retrograde cholangiopancreatography; *F*, female; *M*, male; *PCP*, primary care provider; *UC*, urgent care.

## Discussion

This study suggests that differences occurred between men and women with benign pancreaticobiliary conditions in number of visits or number of days between presentation and ERCP; however, women were less likely to have a complete workup at their first visit. Notably, this variation appears more pronounced in patients who had workup outside the emergency department. This disparity remained even when stratifying for fever, indicating that the greater incidence of fevers seen in men was not the factor driving the differences in diagnostic workup. Importantly, the average age of the men and women was not different, suggesting that age did not significantly contribute to variances. Finally, of the patients who did have laboratory testing at their first visit, there was not a significant difference in proportion of men or women who had elevated bilirubin or alkaline phosphatase.

For almost all diseases, women are diagnosed at a later age and, in most cases, have a longer diagnosis-free interval.^11^ Women have been estimated to spend 25% more of their lives in debilitating health.^12^ A recent cross-sectional analysis of 21.5 million hospital discharges in the United States found that of the 6.2 million cases of infection, the diagnostic error rate in women was 10%, and the serious misdiagnosis-related harm rate was 4.4%.^13^ Although our study was not designed to evaluate whether diagnostic errors contributed to differences in workup, it is worth considering whether bias drives some of the disparities we see in inadequate diagnostic workup at the first visit.

Strengths of this study include that each of the major medical centers involved provided most of the care for patients in their geographic areas. This allowed accurate data to be captured regarding ambulatory and inpatient healthcare visits that patients may have had before ERCP. Additionally, our patient populations were across multiple hospitals in 2 different regions, which may make our results more suggestive of widespread trends. One limitation of our study is the small number of cases evaluated. Larger studies with more racially and ethnically diverse populations are needed to investigate potential delays in care for female patients. Additionally, we were unable to ascertain gender identity and gender expression, focusing instead on sex as our variable of study. Future research should attempt to collect gender identity and expression to understand some of this complexity better.

Our study suggests that women may have inadequate workup for pancreaticobiliary conditions at their first healthcare visit, consistent with the larger body of literature supporting delays in diagnoses. Standardized workups for patients with suspected pancreaticobiliary pathology could help to ensure that implicit or explicit biases do not contribute to inequity in the care for this patient population. Tools that may be helpful in addressing these biases include clinical decision support guidelines and standardized protocols, education of staff, and increased representation of women when creating disparity interventions.^14^ As impressive advancements are being made in the field of gastroenterology, it is important that we continue to collect sex- and gender-disaggregated data to understand, and ultimately address, disparities in outcomes.

## Disclosure

The following authors disclosed financial relationships: D. L. Diehl: Consultant for Boston Scientific, Cook Medical, Pentax, 10.13039/100009734Olympus, GI Supply, Steris Endoscopy, Lumendi, Castle Biosciences, Merit Medical, Acutated Medical, MicroTech, OnePass Medical, Tricol Biomedical, and Kite Endoscopic Innovations. A. Luthra: Consultant for Boston Scientific. S. Mok: Consultant for Steris and Pentax/C2. All other authors disclosed no financial relationships.
